# A scoping review of randomised controlled trials of vaccines that recruited care home residents: lessons for future trials

**DOI:** 10.1093/ageing/afaf355

**Published:** 2025-12-15

**Authors:** Selvarani Subbarayan, Imogen Smith-Dodd, Gabriel Nicolson, Jennifer Kirsty Burton, Janet T Scott, Seshadri S Vasan, Susan D Shenkin, Roy L Soiza, The WATCH Consortium

**Affiliations:** Ageing Clinical and Experimental Research Group, The Institute of Applied Health Sciences, School of Medicine, Medical Sciences and Nutrition, University of Aberdeen, Aberdeen, UK; Aberdeen Royal Infirmary, NHS Grampian, Aberdeen, UK; Ageing Clinical and Experimental Research Group, The Institute of Applied Health Sciences, School of Medicine, Medical Sciences and Nutrition, University of Aberdeen, Aberdeen, UK; Ageing Clinical and Experimental Research Group, The Institute of Applied Health Sciences, School of Medicine, Medical Sciences and Nutrition, University of Aberdeen, Aberdeen, UK; Academic Geriatric Medicine, School of Cardiovascular & Metabolic Health, University of Glasgow, Glasgow, UK; Research, Development and Innovation, NHS Highland, Inverness, UK; School of Medicine, Medical Sciences and Nutrition, University of Aberdeen, Aberdeen, UK; Centre for Virus Research, University of Glasgow, Glasgow, UK; Aberdeen Royal Infirmary, NHS Grampian, Aberdeen, UK; School of Medical and Health Sciences, Edith Cowan University, Joondalup, Australia; Ageing and Health, and Advanced Care Research Centre, Usher Institute, The University of Edinburgh, Edinburgh, UK; Ageing Clinical and Experimental Research Group, The Institute of Applied Health Sciences, School of Medicine, Medical Sciences and Nutrition, University of Aberdeen, Aberdeen, UK; Aberdeen Royal Infirmary, NHS Grampian, Aberdeen, UK

**Keywords:** care home residents, nursing home residents, randomised controlled vaccine trials, recruitment and retention, barriers and facilitators, older people

## Abstract

**Introduction:**

Older care home (CH) residents are particularly vulnerable to infections and often experience adverse outcomes. Despite this group being frequently prioritised for vaccinations, trials of vaccines rarely recruit CH residents. Given that the social and biological characteristics of CH residents may influence vaccine effectiveness, it is crucial to test vaccines in this population.

**Methods:**

The Widening Access to Trials in Care Homes project was established to develop best practice guidance on designing and conducting vaccine trials in the CH population. As part of this project, a scoping review following Joanna Briggs Institute methodology was conducted to identify vaccine trials involving CH residents. Search conducted in EMBASE, MEDLINE, PsycINFO, CINAHL and Cochrane Library, from 1990 to 2025. Results presented as descriptive summaries.

**Results:**

We retrieved 701 articles and included 20 studies. A total of 7479 participants from 238 CHs were recruited to influenza or pneumococcal vaccine trials. The weighted mean age was 82.3 years. Screen failure averaged 70% (eight studies), primarily due to declining participation (46%) and not meeting eligibility criteria (27%). Dropout averaged 8% (11 studies), with death (21%) being the most common reason. Identified barriers include eligibility criteria and recruitment, consent and assent issues, ethical and regulatory, CH-related factors and study time frame and logistical factors. Facilitators include recruitment and data collection methods, consent and assent factors and collaboration with CHs.

**Conclusion:**

Our review is the first to synthesise both quantitative and qualitative evidence on recruiting CH residents into vaccine trials and to provide suggestions for future trial design in this population.

## Key Points

- Exclusion of care home (CH) residents in vaccine trials resulted in a knowledge gap on the safety and efficacy of vaccines.
- Twenty studies reported recruiting CH residents into randomised controlled vaccine trials.
- Our review is the first to summarise the evidence and provide lessons for future vaccine trials involving CH residents.

## Introduction

Older adults living in long-term care facilities (inclusively termed ‘care homes’) are particularly vulnerable to infections, often experiencing significantly higher rates of morbidity and mortality [1, 2]. Around 50% of all COVID-19 related deaths in the UK, Europe and the USA, early in the pandemic occurred in care home (CH) population [3, 4]. Despite this group being prioritised for vaccination [5, 6], CH residents were largely excluded from clinical trials, and none were recruited into COVID-19 vaccine trials [6–10]. The primary reason for exclusion was trial selection criteria. Approximately 60% of vaccine trials had an upper age limit and many excluded individuals with severe comorbidities, cognitive impairment, dementia and frailty [6, 8–10]. Excluding CH residents from vaccine trials has created a knowledge gap on vaccine safety and efficacy in this vulnerable population.

The social and biological characteristics of CH residents may influence vaccine response [6, 8]. Although vaccination reduces infection and mortality rates, real-world studies have reported decreased vaccine effectiveness and rapid waning of immunity in CH residents [11–13], which has been linked to multiple factors, including immunosenescence, frailty, functional impairment, malnutrition, multimorbidity, polypharmacy and a high risk of exposure in communal living settings [1, 12, 14]. Consequently, vaccine efficacy data from community-dwelling older adults may not be directly generalisable to CH residents [1, 6, 11]. Therefore, it is crucial to improve the inclusivity of CH residents in vaccine trials and ensure that trial participants are representative of the ‘real world’ population intended to receive the investigational vaccine following marketing approval [15].

Nevertheless, recruiting CH residents with frailty in vaccine trials presents significant clinical, ethical and logistical challenges [1]. The Enabling Research in Care Homes network in the UK [16] and a similar recent initiative in the USA [17] aim to support research in CHs. However, there is still no established guidance for conducting randomised controlled trials in these settings. To enable the recruitment of CH residents in vaccine trials, the Widening Access to Trials in Care Homes (WATCH) project was established to develop best practice guidance on designing and conducting vaccine trials in the CH population. As part of the WATCH project, we undertook a scoping review with the aim of identifying all published randomised controlled vaccine trials that recruited CH residents and reported barriers and facilitators to successful recruitment and retention.

## Methods

The scoping review was undertaken following the Joanna Briggs Institute methodology for conducting scoping reviews [18, 19]. The protocol was registered on the Open Science Framework (https://osf.io/y64kq) and reported according to the Preferred Reporting Items for Systematic reviews and Meta-Analyses extension for Scoping Reviews (PRISMA-ScR) checklist (Supplementary Appendix 1) [20].

### Search strategy

An iterative process was used to develop a comprehensive search strategy, incorporating the review team expertise, CH setting-specific terms from previous studies [21–23], search filters suggested by The InterTASC Information Specialists’ Sub-Group Search Filter Resource [24] and Cochrane Handbook [25], with librarian input, and guided by the Population, Concept, Context framework.

The database search was conducted on 19 January 2025 by one author (S.S.) across five databases: EMBASE, MEDLINE, PsycINFO, CINAHL and Cochrane Library from 1990 to January 2025. Detailed database search results are provided in Appendix 2 (Supplementary Tables 1–5). No language restrictions were applied; publications not in English were translated either by team members or using Google Translate (https://translate.google.co.uk). Grey literature search was not carried out, however reference and forward citation checking of included articles were performed by two authors (I.S.D. and G.N.). All database results were exported to Rayyan-Intelligent Systematic Review web-tool (https://www.rayyan.ai/) where duplicates were removed before screening articles.

### Inclusion and exclusion criteria

We included commercial and non-commercial vaccine trials that recruited CH residents. All types of randomised trial designs including randomised controlled trials (RCTs), quasi-randomised, cluster randomised trials (CRTs) with individual-level consent and pragmatic RCTs were considered for inclusion. Clinical trial protocols for both ongoing and completed trials were also eligible. Trials were excluded if mean age of residents was <65 years, were non-randomised, non-vaccine and conducted in non-CH settings. Terms used for CH settings (e.g. nursing homes, long-term care facilities and aged CHs) varies across countries. For consistency, we used the term ‘care home’ throughout this review except where alternative terms are used in context.

### Study selection

Three authors (S.S., I.S.D. and G.N.) independently screened titles and abstracts and shortlisted full text articles by consensus. They independently assessed the full text for eligibility. Any disagreements during the study selection process were resolved through discussion with the senior author (R.L.S.).

### Data extraction and analysis

Two reviewers (I.S.D. and G.N.) independently extracted data using a pilot-tested Microsoft Excel sheet, with discrepancies reconciled and verified by a third reviewer (S.S.). Data were extracted on study and participant characteristics, CH details, ethical aspects and funding. Quantitative data on screen failures and dropouts with reasons, as well as qualitative data on recruitment challenges and strategies implemented by investigators to improve recruitment and retention in the vaccine trials were collected. If studies included multiple settings or populations, only data specific to CH residents were extracted. For studies with multiple publications, data were combined to create a comprehensive data for each study. The data extraction form is provided in Appendix 3.

Quantitative data are presented as descriptive summaries and in tables. Qualitative data on recruitment challenges and strategies were first categorised into two broad descriptive themes: barriers and facilitators. These categories were then synthesised into major themes. Qualitative data are reported as descriptive summaries and are also presented in tables.

### Results

**Figure 1:**
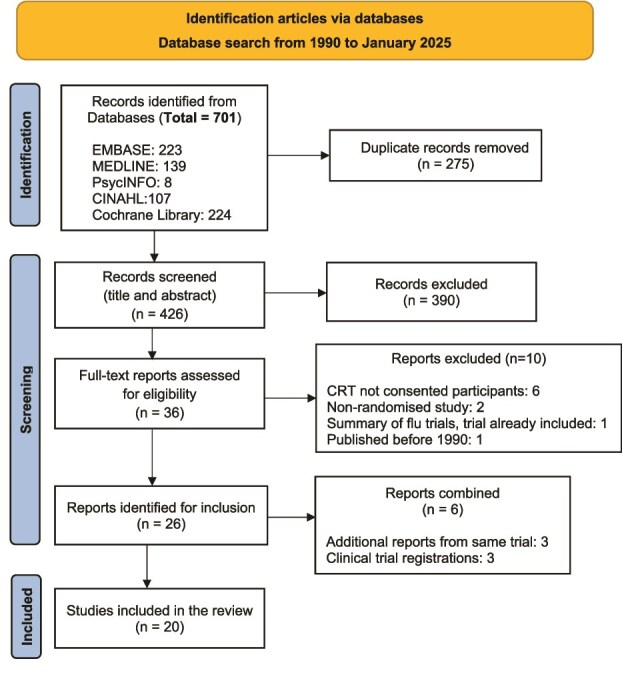
PRISMA flow diagram of the study selection process [54]. CRT: Cluster Randomised Trial.

The database search retrieved 701 records. After removing duplicates, 426 titles and abstracts were screened, and 36 full-text reports were reviewed for eligibility. A total of 26 articles were initially included. However, data from six articles which were additional reports and clinical trial registrations related to the same trials were combined with their main trial publications, resulting in 20 articles included in this scoping review. The PRISMA study flow diagram showing the study selection process is provided in Figure 1.

### Study characteristics

All 20 studies [26–45] were published between 1992 and 2025, with the majority published after 2000. These studies were conducted in 11 different high-income countries [46], mostly from Europe (*N* = 9), followed by America (*N* = 6) and Asia (*N* = 5). All were published in English except one in French. The majority (*N* = 17) tested influenza vaccines; three [39, 41, 43] assessed pneumococcal vaccines. Follow-up period, reported in 15 studies, ranged from 4 weeks to 26 months. Most studies (*N* = 13) had a follow-up period of less than seven months, and only two studies [29, 31] had a longer follow-up of approximately two years. Of the 20 studies, 10 evaluated unlicensed vaccines while the other 10 tested licensed vaccines. Response to vaccine (immunogenicity) was reported as the main outcome in 18 studies. Although five studies reported clinical efficacy outcomes, this was the primary outcome only in one study [41].

### Participant characteristics

7479 older people living with frailty from 238 CHs, residential homes or long-term care facilities were recruited in 20 trials of influenza or pneumococcal vaccines. All included trials recruited only CH residents, except two [27, 28], which enrolled both CH residents and younger volunteers. However, only data relevant to CH residents are presented in this review. Sample size ranged from 43 to 1959 (median = 270) with the majority of trials (*N* = 12, 60%) recruiting less than 300 participants. The weighted mean age from 18 studies was 82.3 years and most of the studies had ≥50% females. Ethnicity was reported in only three studies [40, 44, 45] which showed that the majority were white (ranging from 81.9% to 98.9%), with a minority of participants from black (12.4%), and other ethnicities ranging from 1.1% to 5.7% [44, 45]. A summary of the study and participant characteristics of the included studies is presented in Table 1, with detailed study characteristics provided in Appendix 4.

**Table 1:** Summary characteristics of included vaccine trials.

Author	Country	Study design	Disease condition	Study period	Follow-up period	CHs (*N*)	Sample size	Mean age (years)	Female *n* (%)
Treanor *et al.* [26]	USA	Randomised, double-blind, placebo-controlled study	Influenza	1987–1990	NR	3	523	84.2	75%
Remarque *et al.* [27]	Netherlands	Extension study of Randomised, double- blind study	Influenza	NR	NR	NR	79^a^	81^a^	NR
Palache *et al.* [28]	Netherlands, France, Israel	Randomised, double blind, placebo-controlled, multicentre dose response study	Influenza	October–December 1988	NR	21	262^a^	80^a^	NR
Glück *et al.* [29]	Italy	Randomised study	Influenza	NR	4 weeks	1	126	78	88 (70%)
Gravenstein *et al.* [30]	USA	Randomised, prospective, double-blind trial	Influenza	NR	5 months	14	408	79.4 ± 9.0	168 (42.3%)
Gorse *et al.* [31]	USA	Randomised, double blind, placebo-controlled study	Influenza	May 1993–June 1994	4 weeks	2	50	74.9 ± 1.2	7 (14%)
Gauthey *et al.* [32]	Switzerland	Randomised study	Influenza	NR	NR	51	1959	84 ± 8	1524 (77.8%)
Rudenko *et al.* [33]	Russia	Randomised, placebo-controlled, double-blind study	Influenza	1996–1997 influenza season	NR	9	614	70.5 ± 8.8	70%
Roos-van Eijndhoven *et al.* [34]	Netherlands	Randomised controlled trial	Influenza	1997–1998 influenza season	3.6 months	14	815	82.9 ± 1.7	74.8%
Pregliasco *et al.* [35]	Italy	Prospective, observer-blind, randomised, multicentre trial	Influenza	1998–1999	7 months	4	635	85	498 (78.4%)
Baldo *et al.* [36]	Italy	Randomised, double-blind trial	Influenza	1998–1999 winter season	4 weeks	NR	300	83.3	215 (75.4%)
Ben-Yehuda *et al.* [37]	Israel	Randomised, double-blind trial	Influenza	Winter of 2000–2001	6 months	NR	81	81.3 ± 7.8	55 (67.9%)
Muszkat *et al.* [38]	Israel	Clinical trial	Influenza	1998	6 weeks	NR	43	77.5 ± 13	26 (60%)
Valenzuela *et al.* [39]	Chile	Randomised double-blind controlled study	Pneumococcal disease	August 1998–September 2000	2 years	1	118	NR	53 (44.9%)
Gaughran *et al.* [32]	UK	Randomised, multicentre, observer-blind, parallel group, controlled trial	Influenza	Aug 2004–April 2005	7 months	26	277	NR	184 (69.9%)
Maruyama *et al.* [41]	Japan	Randomised, prospective, multicentre, double blind, placebo-controlled study	Pneumococcal pneumonia	March 2006–March 2009	26 months	23	1006	84.8 ± 7.7	786 (78.1%)
Chan *et al.* [42]	Hong Kong SAR, China	Randomised, prospective, open-label, single-centre, parallel group, controlled trial (Phase 4)	Influenza	October 2013–April 2014	6 months	9	100	82.9 ± 7.4	63 (63%)
Namkoong *et al.* [43]	Japan	Randomised, prospective, open-label study	Pneumococcal disease	April 2011–December 2012	1 month	5	105	88 ± 1.48	77 (77%)
Nace *et al.* [44]	USA	Randomised, single-blinded, controlled trial (Phase 4 trial)	Influenza	September 2011–March 2013	6 months	15	205	86.7 ± 6	128 (68.4%)
Didion *et al.* [45]	USA	Randomised active-controlled, non-inferiority trial (Phase 4 trial)	Influenza	2018–2019 & 2019–2020 (two influenza seasons)	6 months	40	387	80.1 ± 9.6	195 (50.6%)

^a^The results are presented only for older residents, but the trial included both nursing home residents and young volunteers. CHs: Care Homes; NR: Not Reported.

### Eligibility criteria and recruitment method

Among the 20 included studies, three failed to report study eligibility criteria. Six studies [28, 30, 31, 33, 41, 42] excluded participants with impaired memory and dementia who were unable to provide informed consent, while one study [40] excluded residents with current evidence of delirium. Two studies [33, 44] excluded severely debilitated and terminally ill residents or residents with a life expectancy of <6 months and five studies [29, 38, 39, 42, 45] excluded residents with chronic medical conditions. Additionally, many studies excluded immunocompromised residents due to underlying illness or immunosuppressive therapy.

Only four studies [34–36, 44] reported methods of participant recruitment which varied widely: consecutive enrolment [36], random recruitment [35] or invitation by mail [34]. Nace *et al.* [44] used several different recruitment methods, including informational flyers, social gatherings and facility staff referrals. Additionally, a letter introducing the study was sent to potential subjects by the CH administration.

### Screen failure and dropouts

Screening details were reported only in eight studies [30, 34, 40–45], conducted in the USA (*N* = 3), Japan (*N* = 2), UK (*N* = 1), Netherlands (*N* = 1) and Hong Kong SAR (*N* = 1), and published between 1994 and 2025. All investigated influenza vaccines except two Japanese studies that evaluated pneumococcal vaccines. Overall, 11 356 CH residents were screened and 7911 (69.7%) were screen failures. The screen failure rate ranged from 19.0% to 90.0%, and a high screen failure rate of ≥65.0% was reported in six of the eight studies. In contrast, two studies [41, 45] from Japan (2015) and the USA (2025,) reported lower screen failure rates of 30% and 19%, respectively.

Of eight studies that reported data on screening, seven [30, 40–45] provided reasons for screen failure. The two most common reasons for screen failure were residents declining participation (*n* = 3628, 45.9%) and not meeting the eligibility criteria (*n* = 2127, 26.9%). These remained the main reasons for screen failure even in studies with lower screen failure rates. The dropout rate from 11 studies was 7.6% (*n* = 380). Death (*n* = 80, 21.1%) was the most common reason for dropout, reported in five studies. Data on screen failure and dropout rates and reasons for the same are provided in Tables 2 and 3, respectively.

**Table 2:** Screen failure and dropouts.

Author	Total screened (*n*)	Total recruited (*n*)	Total screen failure *n* (%)	Declined participation *n* (%)	Did not meet study criteria *n* (%)	Dropouts *n* (%)	Dropouts due to death *n* (%)
Gravenstein *et al.* [30]	2594	408	2186 (84.3%)	975 (44.6%)	1229 (56.2%)	11 (2.7%)	7 (63.6%)
Gaughran *et al.* [40]	968	277	691 (71.4%)	225 (32.6%)	90 (13.0%)	2 (0.72%)	2 (100%)
Maruyama *et al.* [41]	1434	1006	428 (29.8%)	395 (92.3%)	18 (4.2%)	None	169^a^
Chan *et al.* [42]	703	100^b^	461 (65.6%)	47 (10.2%)	362 (78.5%)	4 (4.0%)	NR
Namkoong *et al.* [43]	623	105	518 (83.1%)	151 (29.2%)	365 (70.5%)	5 (4.8%)	1 (20%)
Nace *et al.* [44]	2112	205	1907 (90.3%)	1795 (94.1%)	12 (0.63%)	18 (8.8%)	11^a^
Didion *et al.* [45]	478	387	91 (19.0%)	40 (44.0%)	51 (56.0%)	18 (4.7%)	NR
Roos-van Eijndhoven *et al.* [34]	2444	815	1629 (66.7%)	NR	NR	191 (23.4%)	69 (36.1%)
Rudenko *et al.* [33]	NR	614	NR	NR	NR	19 (3.1%)	NR
Pregliasco *et al.* [35]	NR	635	NR	NR	NR	16 (2.5%)	1 (6.3%)
Baldo *et al.* [36]	NR	300	NR	NR	NR	15 (5.0%)	NR
Valenzuela *et al.* [39]	NR	118	NR	NR	NR	81 (68.6%)	NR
**Total**	11 356	4970	7911 (69.7%)	3628 (45.9%)	2127 (26.9%)	380 (7.6%)	80 (21.1%)

^a^Not included in the dropout % calculation as death was not reported as the reason for dropout and these participants were included in the study analysis.

^b^Recruited 100 from 242 suitable residents, no further information available. NR: Not Reported.

**Table 3:** Reasons for screen failure and dropouts.

Reasons for screen failure	Reasons for dropout
Residents declined to participate (45.9%)^a^ Did not meet the study inclusion criteria (26.9%)^a^ Unable to reach relatives for consent (39.7%)^c^ Relatives declined Refused vaccination Already vaccinated CH staff declined consent General Practitioner declined consent Residents not available Hospitalised/left CH Other reasons	**Dropout during follow-up** Died during follow-up (21.1%)^b^ Withdrew consent Missing or insufficient blood samples for laboratory analysis (immune response) Lost to follow-up Left CH facility Hospitalised/transferred to other facility Not present during follow-up visit Other reasons **Dropout at randomisation visit** Died before randomisation (didn’t receive study vaccine) Found not meeting eligibility criteria Vaccine administered by non-clinical team or blinding broken Pre-vaccination blood samples missed

^a^Data from seven studies.

^b^Data from five studies.

^c^Data from one study. Authors were not able to calculate the % for all the reasons due to non-availability of data in all included studies.

### Care home characteristics

Of the 20 studies, four [27, 36–38] did not report on the number of CHs involved; the remaining 16 studies [26, 28–35, 39–45] recruited from 238 CHs. The number of CHs involved in recruitment ranged from 1 to 51 (Table 1). Regarding the types of CHs, 17 studies recruited from nursing homes, and two studies [26, 40] recruited from long-term care facilities that included both nursing homes and residential homes. One study [44] recruited from different long-term care facilities, including nursing homes, assisted-living or personal CHs and independent-living facilities.

The type of services provided by CHs were reported only in two studies [26, 40] which stated that CHs provided both nursing care and residential care services. CH size (numbers of beds) was only reported two studies [34, 44]. Roos-van Eijndhoven *et al.* [34] recruited from 14 nursing homes with 60 to 320 beds and Nace *et al.* [44] from 15 with 42 to 178 beds. None of the studies reported whether the CH were public or privately owned.

### Ethical and regulatory aspects

All 20 studies obtained informed consent. Interestingly, Gauthey *et al.* [32] that tested routine seasonal influenza vaccine reported obtaining verbal consent for vaccination and study participation. In four studies [34, 40, 44, 45], consent was obtained from participants or their legally authorised representatives (LAR) who are either relatives, CH staff or healthcare professionals for people unable to consent. Three studies [26, 28, 41], obtained consent from participants or their next of kin. The remaining 13 did not specify proxy consent procedures; six of these excluded individuals lacking capacity.

Three studies reported how the resident’s capacity to consent was assessed. Two studies [26, 45] reported that the study team, with input from CH staff, determined whether informed consent from the participant or LAR was required based on residents’ decision-making ability regarding their medical care. Didion *et al.* [45] reported that after consulting CH staff, the study team also ascertained residents understanding of the trial during consent by asking several questions. Gaughran *et al.* [40] reported that research nurses were trained in the Mini-Mental State Examination, Cornell Scale for Depression in Dementia and capacity assessments.

Ethics committee approval was reported in 14 studies. One study [31] did not explicitly mention ethics approval, although stated adherence to the guidelines of the human studies subcommittee and the institutional review board. The remaining five studies did not report whether ethical approval was obtained. All five, published between 1994 and 2001, evaluated influenza vaccines and were conducted in the USA, Switzerland, Russia and Italy (*N* = 2).

One study [45] provided financial incentives to participants for their participation and additional payments for completing blood sample collection. The same study also offered free influenza vaccines to CH employees and provided stipends to participating facilities to cover administrative costs associated with staff involvement in trial recruitment and data collection. No other study reported offering incentives or payments for trial participation.

### Trial registration and funding

Of the 20 studies, only five were registered on clinical trials registries; 15 studies did not report trial registration details. Funding sources were not disclosed in eight studies. Although a few studies received support or partial funding from the pharmaceutical industry, all 20 included studies were investigator-led, and none were pharmaceutical-sponsored trials.

### Qualitative evidence on barriers to recruiting care home residents

Of the 20 studies, only six trials [30, 40–42, 44, 45] reported on the recruitment challenges experienced by the investigators during the vaccine trial conduct and were categorised into five major themes (Table 4).

**Table 4:** Barriers and facilitators identified from vaccine trials in CH residents.

Major themes	Barriers reported	Reported by
Eligibility criteria and recruitment	Exclusion of CH residents with cognitive impairment Challenges in recruiting frail older adults: very low recruitment and retention rate	[42, 44]
Consent and assent issues	Unable to contact relatives in time for consenting Translated consent forms not available to recruit non-English speaking residents	[40, 44, 45]
Ethical and regulatory factors	IRB protection of vulnerable CH population Placebo-controlled trial deemed unethical	[30, 41, 44]
CH-related factors	Gatekeeper barriers: Unable to reach CHs and objection from CH management & General Practitioner The CH infrastructure present challenges	[40, 42]
Study time frame and logistical factors	Short recruitment period for vaccine trials, which competes with the seasonal immunisation programme of CHs Logistical issues delayed recruitment and vaccination.	[40, 44]
	**Facilitators reported**	
Recruitment and data collection methods	Inclusion of CH residents with cognitive impairment Improved communication: In-person meetings at CHs to inform residents about the benefits and risks of trials Recruitment from multiple CHs. Change in study procedure to improve recruitment Use multiple methods (involve healthcare professionals, CH staff, carers) to collect data on outcomes and adverse events. Minimise withdrawals by identifying residents who are likely to stay longer in the facility to complete the study follow up.	[33, 40, 41, 43–45]
Consent and assent factors	Contact with relatives well in advance for consenting allowing lead-in time Collect contact details of the second family member to approach if required for consent process.	[40, 45]
Collaboration with CHs	Agreement with CH to work collaboratively for participant recruitment and trial conduct	[45]

CH: Care Home; IRB: Institutional Review Board.

#### Eligibility criteria and recruitment

Chan *et al.* [42] reported that their study excluded residents with cognitive impairment which led to increased screen failure, and limited generalisability of the results in this population. Nace *et al.* [44] reported that the recruitment rate in their trial was only 9.7%. Although low recruitment rates are expected in the CH population, this rate was lower than in clinical trials in other settings and highlighted the challenges of recruiting and retaining frail older adults.

#### Consent and assent issues

Nace *et al.* [44] and Gaughran *et al.* [40] reported excluding potential subjects due to difficulties reaching relatives in time for proxy consent. Gaughran *et al.* [40] added that this led to higher recruitment of residents who had the capacity to consent resulting in a study sample that is relatively healthier. Didion *et al.* [45] reported excluding non-English speakers due to budget constraints that limited translation of consent forms into multiple languages, though the trial team indicated such exclusion is rare in their experience.

#### Ethical and regulatory factors

Gravenstein *et al.* [30] and Nace *et al.* [44] reported that institutional review boards ‘protected vulnerable individuals’ and refused vaccine trials involving CH residents lacking capacity to consent. Maruyama *et al.* [41] mentioned that a placebo-controlled trial of vaccine efficacy was considered unethical, especially in high-income countries where vaccination is standard of care, despite limited efficacy data.

#### Care home-related factors

Gaughran *et al.* [40] reported difficulties in contacting CHs. In some CHs, the CH management and the General Practitioner declined participation in vaccine trials. Chan *et al.* [42] reported that the unique characteristics of CH infrastructure presented significant challenges to recruiting CH residents.

#### Study time frame and logistical factors

Gaughran *et al.* [40] reported logistical issues delayed recruitment and 60% of participants missed booster vaccinations within 3 weeks of randomisation. Nace *et al.* [44] reported a very short recruitment window, which overlapped with CH residents’ seasonal immunisation programmes that vaccinate all residents within 48 hours, made it challenging to enrol and vaccinate participants before routine vaccinations began.

### Qualitative evidence on facilitators to recruiting care home residents

Of the 20 studies, six reported on the facilitators and strategies used by the trial team to improve recruitment and trial conduct. The facilitators reported from six [33, 40, 41, 43–45] vaccine trials were categorised into three major themes and are described in Table 4.

#### Recruitment and data collection methods

Rudenko *et al.* [33] held meetings at CHs to explain the study purpose, potential benefits, risks and discomfort. Nace *et al.* [44] recruited from multiple CHs and included residents with significant cognitive impairment to improve recruitment. Didion *et al.* [45] minimised withdrawal by selecting participants who are likely to stay longer in the same facility to complete follow up visits. Gaughran *et al.* [40] improved recruitment by removing the requirement for pre-vaccination blood samples. Three studies [41, 43, 45] reported using various data collection methods, including monitoring for adverse events by the medial, research or CH team by regular and close follow-up visits, regular medical team follow up to collect data on clinical outcomes and requesting relatives to report adverse events to the trial team.

#### Consent and assent factors

Gaughran *et al.* [40] recommended contacting relatives well in advance allowing a longer lead in time for the project to obtain consent from relatives. Didion *et al.* [45] collected the contact details of the second family relative where required to approach for the consent process.

#### Collaboration with care homes

Didion *et al.* [45] reported making a formal agreement with CH management and staff and working collaboratively to carry out trial activities including study advertisement, screening, consent, recruitment and data collection.

**Table 5:** Suggestions for future vaccine trial design in CH residents.

Trial phase	Suggestions for vaccine trial design in CH residents
Protocol development	Broaden inclusion criteria to include residents who are unable to consent and or with multiple chronic conditions. Select outcomes that are appropriate to CH residents. Calculate the sample size considering the high screen failure and dropout rates. Assess factors to reduce screen failure and minimise dropouts. Plan recruitment from multiple CHs to improve the recruitment rate Promote diversity in the population recruited by including ethnic minorities and non-English speaking residents.
Trial recruitment	Establish good communication and explain the risk-benefits of the vaccines to residents and/or relatives. Contact relatives in advance to obtain consent with a lead in time in the project planning particularly for trials with shorter recruitment period
Data collection	Reduce the burden on participants for collecting data by reducing the number of blood samples and/or employing alternative data collection methods. Multiple sources of data collection methods: involve study team, healthcare team, CH staff and relatives or carers for collecting outcome data.
Trial management	Efficient trial planning to avoid logistical delays that affect recruitment and vaccination
Ethics and Regulatory discussion	Discuss with ethics committees and regulatory bodies to include CH residents in vaccine trials
Collaboration with CH-related partners	Make formal agreements with CHs to work collaboratively and involve the healthcare team to reduce gatekeeper barriers. Consider involving experts in CH research and the healthcare team providing medical care to the CH residents.

CH: Care Home.

## Discussion

This is the first review, to the best of our knowledge, to map and summarise the evidence on randomised controlled vaccine trials conducted in the CH population. Most importantly, we found quantitative evidence on screen failure and dropout reported in vaccine trials along with qualitative evidence on certain barriers and facilitators to recruiting CH residents. Gordon *et al.* reported on randomised controlled trials in CHs from a systematic mapping review [11]. That review included a wide range of pharmacological and non-pharmacological interventions and different types of trial design whereas our review is focused on randomised controlled vaccine trials.

We found a high screen failure rate of around 70% in the CH residents from eight vaccine trials. The two most common reasons for the screen failure were residents declining study participation and not meeting eligibility criteria. Notably, two studies [41, 44] conducted in Japan and the USA reported around 90% declined to participate in the study. Moreover, Gaughran *et al.* [30] (UK) reported that 78.4% of the screen failures were due to consent/assent issues. Although, cognitive impairment was not explicitly reported as a reason for screening failure, it was likely included under the category of ‘not meeting the eligibility criteria’ as most of these studies excluded individuals with cognitive impairment and dementia. While some eligibility criteria were directly applicable to vaccine efficacy and safety (e.g. immunocompromised), many trials excluded individuals with severe chronic illness, cognitive impairment, dementia and frailty, and these conditions are highly prevalent in the CH population [47]. This shows the importance of making inclusion and exclusion criteria as permissive as possible to understand vaccine efficacy in this population. The high screen failure rate needs to be factored into sample size calculations. Allowing extra time to engage family members and other healthcare professionals involved in the residents’ care may improve study participant. Specific guidance for involving those who lack the capacity to consent is needed to facilitate inclusive research [48].

We identified a relatively low (8%) dropout rate in CH residents, nevertheless the follow-up period in most of the studies was very short. The commonest reason for dropout was death (21%). Higher mortality is a recognised challenge when undertaking research involving this population and the average life expectancy of CH residents in Scotland is 2 to 2.5 years [49]. Participants withdrawing consent and missing or unsuccessful post-vaccination blood samples were also reported as common reasons for dropout. This emphasises that blood withdrawal might be difficult in frail, older CH residents. Ensuring the team includes an experienced phlebotomist and/or reducing the number of blood samples will be important in future trial design.

There is no literature evidence available to compare our findings on screen failure and dropout rates. If future vaccine trials report more information on the recruitment issues, it will help the researchers to identify the challenges and potential solutions to recruitment and retention of CH residents into vaccine trials. All the included vaccine trials were investigator-led trials, and none were conducted directly by the pharmaceutical companies though a few trials were supported by the pharmaceutical companies. This highlights the longstanding exclusion of CH residents from pharmaceutical company-sponsored randomised controlled vaccine trials.

Our review identified many practical recruitment challenges and strategies used by investigators in their vaccine trials (Table 4). Moreover, Bath *et al.* [50] reported various challenges in conducting trials within the CH setting, based on lessons learned from setting up the PROTECT-CH COVID-19 platform trial in UK CHs. When planning vaccine trials in older CH residents with complex needs, it is important that researchers consider the various recruitment and retention issues highlighted in our review. Based on our findings, we have provided suggestions for planning future vaccine trials in the CH population in Table 5.

Greater cooperation between the vaccine research and development community, the CH research community and those with lived experience in CH settings should help successful recruitment of this under-represented group in the future vaccine trials. The success in researcher-led trials ought to be reproducible in commercial trials to avoid a repetition of the important evidence gap seen during the COVID-19 pandemic. There are some encouraging signs that pharmaceutical companies are trying to recruit CH residents. A recent 2025 report from a Phase 3 trial [51] that tested the respiratory syncytial virus vaccine in 17 countries reported that they successfully recruited 307 residents from long-term care facilities, representing 1.2% of the total trial population. The inclusion and diversity plans introduced recently by regulatory authorities in the UK, Europe and the USA to improve representation of under-represented populations in clinical trials is another positive step toward the inclusion of CH residents in vaccine trials [7, 15, 52, 53].

### Strengths and limitations

This is the first review to report both quantitative and qualitative evidence on barriers and facilitators to recruiting CH residents to randomised controlled vaccine trials. The evidence generated is from reports of actual experiences and observations from vaccine trials conducted in care home residents. Another strength is that we followed the methodology recommended by the Joanna Briggs Institute for conducting the scoping review. Moreover, the search strategy and protocol were developed following an iterative process among the authors and input from the WATCH project Advisory Group members who are experts in vaccine trials, CH research, infectious diseases and geriatricians involved in the clinical care of older people.

Some limitations are acknowledged. Firstly, we excluded CRTs of vaccines that did not gain consent at the resident level (i.e. randomised by CH). It is possible that some additional learning from these was missed, but this design is not applicable to trials of investigational vaccines that are conducted to obtain marketing approval. Secondly, seven out of 20 included trials were published before 2000, which may not well reflect current practices in either CH management or trial conduct. Nevertheless, their inclusion provided useful data. Finally, quality of reporting was variable, limiting what could usefully be extracted from trials.

## Conclusion

Our scoping review is the first to report screen failure and dropout rate, and several major themes on barriers and facilitators to successful recruitment and retention of older CH residents into randomised controlled trials of vaccines. We identified strategies that will facilitate the recruitment of CH residents into future vaccine trials. This will in turn allow CH residents, their carers, healthcare professionals, healthcare providers and policy makers to make better, evidence-based decisions around vaccination.

## Supplementary Material

Supplementary_data_afaf355
