# Supplementary material for: A scoping review of randomised controlled trials of vaccines that recruited care home residents: lessons for future trials
Source: Age Ageing. 2025 Dec 15;54(12):afaf355. doi: 10.1093/ageing/afaf355 (PMC12704422; doi:10.1093/ageing/afaf355)
Supplement: Supplementary_data_afaf355 [file supplementary_data_afaf355.docx]

**Supplementary Data**

**Title:** A scoping review of randomised controlled trials of vaccines that recruited care home residents: lessons for future trials

**Contents**

[Appendix 1: Preferred Reporting Items for Systematic reviews and Meta-Analyses extension for Scoping Reviews (PRISMA-ScR) Checklist 1](#_Toc216098041)

[Appendix 2: Database search results 3](#_Toc216098042)

[Supplementary Table 1. Search results from EMBASE (Ovid) 3](#_Toc216098043)

[Supplementary Table 2. Search results from MEDLINE (Ovid) 5](#_Toc216098044)

[Supplementary Table 3. Search results from PsycINFO (Ovid) 6](#_Toc216098045)

[Supplementary Table 4. Search results from CINAHL (EBSCOhost) 8](#_Toc216098046)

[Supplementary Table 5. Search results from Cochrane Library 9](#_Toc216098047)

[Appendix 3: Data Extraction Form 11](#_Toc216098048)

[Appendix 4: Detailed study characteristics of included vaccine trials 13](#_Toc216098049)

# Appendix 1: Preferred Reporting Items for Systematic reviews and Meta-Analyses extension for Scoping Reviews (PRISMA-ScR) Checklist

| **SECTION** | **ITEM** | **PRISMA-ScR CHECKLIST ITEM** | **REPORTED ON PAGE #** |
| --- | --- | --- | --- |
| **TITLE** | | | |
| Title | 1 | Identify the report as a scoping review. | 1 |
| **ABSTRACT** | | | |
| Structured summary | 2 | Provide a structured summary that includes (as applicable): background, objectives, eligibility criteria, sources of evidence, charting methods, results, and conclusions that relate to the review questions and objectives. | 1 to 2 |
| **INTRODUCTION** | | | |
| Rationale | 3 | Describe the rationale for the review in the context of what is already known. Explain why the review questions/objectives lend themselves to a scoping review approach. | 2 |
| Objectives | 4 | Provide an explicit statement of the questions and objectives being addressed with reference to their key elements (e.g., population or participants, concepts, and context) or other relevant key elements used to conceptualize the review questions and/or objectives. | 2 |
| **METHODS** | | | |
| Protocol and registration | 5 | Indicate whether a review protocol exists; state if and where it can be accessed (e.g., a Web address); and if available, provide registration information, including the registration number. | 2 |
| Eligibility criteria | 6 | Specify characteristics of the sources of evidence used as eligibility criteria (e.g., years considered, language, and publication status), and provide a rationale. | 3 |
| Information sources | 7 | Describe all information sources in the search (e.g., databases with dates of coverage and contact with authors to identify additional sources), as well as the date the most recent search was executed. | 3 |
| Search | 8 | Present the full electronic search strategy for at least 1 database, including any limits used, such that it could be repeated. | 2 to 3 & Appendix 2 |
| Selection of sources of evidence | 9 | State the process for selecting sources of evidence (i.e., screening and eligibility) included in the scoping review. | 3 |
| Data charting process | 10 | Describe the methods of charting data from the included sources of evidence (e.g., calibrated forms or forms that have been tested by the team before their use, and whether data charting was done independently or in duplicate) and any processes for obtaining and confirming data from investigators. | 3 |
| Data items | 11 | List and define all variables for which data were sought and any assumptions and simplifications made. | 3 & Appendix 3 |
| Critical appraisal of individual sources of evidence | 12 | If done, provide a rationale for conducting a critical appraisal of included sources of evidence; describe the methods used and how this information was used in any data synthesis (if appropriate). | Not done |
| Synthesis of results | 13 | Describe the methods of handling and summarizing the data that were charted. | 3 |
| **RESULTS** | | | |
| Selection of sources of evidence | 14 | Give numbers of sources of evidence screened, assessed for eligibility, and included in the review, with reasons for exclusions at each stage, ideally using a flow diagram. | 3 & Figure 1 on page 4 |
| Characteristics of sources of evidence | 15 | For each source of evidence, present characteristics for which data were charted and provide the citations. | 3, 5 & Appendix 4 |
| Critical appraisal within sources of evidence | 16 | If done, present data on critical appraisal of included sources of evidence (see item 12). | Not done |
| Results of individual sources of evidence | 17 | For each included source of evidence, present the relevant data that were charted that relate to the review questions and objectives. | 3 to 6 |
| Synthesis of results | 18 | Summarize and/or present the charting results as they relate to the review questions and objectives. | 4 to 9 |
| **DISCUSSION** | | | |
| Summary of evidence | 19 | Summarize the main results (including an overview of concepts, themes, and types of evidence available), link to the review questions and objectives, and consider the relevance to key groups. | 8-10 |
| Limitations | 20 | Discuss the limitations of the scoping review process. | 10 |
| Conclusions | 21 | Provide a general interpretation of the results with respect to the review questions and objectives, as well as potential implications and/or next steps. | 10 |
| **FUNDING** | | | |
| Funding | 22 | Describe sources of funding for the included sources of evidence, as well as sources of funding for the scoping review. Describe the role of the funders of the scoping review. | 10 |

# Appendix 2: Database search results

## Supplementary Table 1. Search results from EMBASE (Ovid)

| **[#](https://ovidsp.dc1.ovid.com/ovid-new-b/ovidweb.cgi?&S=IKPLFPBKLKACCHMAKPIJMFEMMHPLAA00&Sort+Sets=descending)** | **Search terms** | **Results** |
| --- | --- | --- |
| 1 | exp aged/ | 4189068 |
| 2 | exp aging/ | 367466 |
| 3 | ageing.tw. | 77320 |
| 4 | elder*.tw. | 467312 |
| 5 | exp frail elderly/ | 12945 |
| 6 | older people.tw. | 51287 |
| 7 | older adult*.tw. | 172691 |
| 8 | older population.tw. | 10631 |
| 9 | older individual*.tw. | 18319 |
| 10 | oldest old.tw. | 4169 |
| 11 | exp geriatrics/ | 61926 |
| 12 | geriatric*.tw. | 107176 |
| 13 | very elderly/ | 334206 |
| 14 | randomized controlled trial/ | 862368 |
| 15 | randomi?ed controlled trial.tw. | 199386 |
| 16 | RCT.tw. | 60921 |
| 17 | controlled clinical trial/ | 442539 |
| 18 | controlled trial.tw. | 254468 |
| 19 | clinical trial/ | 1106455 |
| 20 | trial*.tw. | 2091656 |
| 21 | clinical study/ | 181278 |
| 22 | randomi?ed.tw. | 1245802 |
| 23 | randomization/ | 99780 |
| 24 | randomi?ation.tw. | 95760 |
| 25 | random allocation.tw. | 2834 |
| 26 | randomly allocated.tw. | 49545 |
| 27 | random assignment.tw. | 3553 |
| 28 | care home*.tw. | 8089 |
| 29 | nursing home/ | 68598 |
| 30 | old age home*.tw. | 563 |
| 31 | aged care home*.tw. | 225 |
| 32 | residential home/ | 8643 |
| 33 | residential care.tw. | 5670 |
| 34 | residential facilit*.tw. | 1555 |
| 35 | long term care.tw. | 33840 |
| 36 | long-term care setting*.tw. | 2087 |
| 37 | long-term care facilit*.tw. | 9709 |
| 38 | care home setting*.tw. | 317 |
| 39 | home for the aged/ | 13919 |
| 40 | aged care facilit*.tw. | 1877 |
| 41 | institutional care/ | 6912 |
| 42 | institutionalization/ | 9999 |
| 43 | institutionali?ation.tw. | 8208 |
| 44 | institutionalized adult/ or institutionalized elderly/ | 470 |
| 45 | institutionali?ed.tw. | 16966 |
| 46 | vaccine trials.tw. | 3534 |
| 47 | vaccine trial.tw. | 2610 |
| 48 | vaccine study.tw. | 613 |
| 49 | vaccine studies.tw. | 1504 |
| 50 | vaccine/ | 91862 |
| 51 | vaccin*.tw. | 565621 |
| 52 | vaccination/ | 260464 |
| 53 | 1 or 2 or 3 or 4 or 5 or 6 or 7 or 8 or 9 or 10 or 11 or 12 or 13 | 4629548 |
| 54 | 14 or 15 or 16 or 17 or 18 or 19 or 20 or 21 or 22 or 23 or 24 or 25 or 26 or 27 | 3431902 |
| 55 | 28 or 29 or 30 or 31 or 32 or 33 or 34 or 35 or 36 or 37 or 38 or 39 or 40 or 41 or 42 or 43 or 44 or 45 | 146251 |
| 56 | 46 or 47 or 48 or 49 or 50 or 51 or 52 | 624721 |
| 57 | 53 and 54 and 55 and 56 | 228 |
| **58** | **limit 57 to yr="1990 -Current"** | **223** |

## Supplementary Table 2. Search results from MEDLINE (Ovid)

| **#** | **Search terms** | **Results** |
| --- | --- | --- |
| 1 | exp Aged/ | 3629201 |
| 2 | exp Aging/ | 305511 |
| 3 | ageing.tw. | 57061 |
| 4 | elder*.tw. | 323818 |
| 5 | exp Frail Elderly/ | 16840 |
| 6 | older people.tw. | 41609 |
| 7 | older adult*.tw. | 141319 |
| 8 | older population.tw. | 7675 |
| 9 | older individual*.tw. | 14493 |
| 10 | oldest old.tw. | 3291 |
| 11 | exp Geriatrics/ | 32069 |
| 12 | geriatric*.tw. | 64227 |
| 13 | "Aged, 80 and over"/ | 1059115 |
| 14 | Randomized Controlled Trial/ | 630208 |
| 15 | randomi?ed controlled trial.tw. | 156081 |
| 16 | RCT.tw. | 36753 |
| 17 | Controlled Clinical Trial/ | 95676 |
| 18 | controlled trial.tw. | 195456 |
| 19 | Clinical Trial/ | 540985 |
| 20 | trial*.tw. | 1462185 |
| 21 | Clinical Study/ | 6504 |
| 22 | randomi?ed.tw. | 876249 |
| 23 | randomi?ation.tw. | 63661 |
| 24 | Random Allocation/ | 107993 |
| 25 | randomly allocated.tw. | 40403 |
| 26 | random assignment.tw. | 2973 |
| 27 | care home*.tw. | 6072 |
| 28 | Nursing Homes/ | 40865 |
| 29 | old age home*.tw. | 367 |
| 30 | aged care home*.tw. | 204 |
| 31 | residential home*.tw. | 1102 |
| 32 | residential care.tw. | 4573 |
| 33 | Residential Facilities/ | 5810 |
| 34 | residential facilit*.tw. | 1214 |
| 35 | Long-Term Care/ | 29392 |
| 36 | long-term care setting*.tw. | 1697 |
| 37 | long-term care facilit*.tw. | 7551 |
| 38 | care home setting*.tw. | 202 |
| 39 | Homes for the Aged/ | 15245 |
| 40 | aged care facilit*.tw. | 1661 |
| 41 | institutional care.tw. | 2348 |
| 42 | Institutionalization/ | 5577 |
| 43 | institutionali?ation.tw. | 6005 |
| 44 | institutionali?ed.tw. | 12573 |
| 45 | vaccine trial.tw. | 1987 |
| 46 | vaccine trials.tw. | 2939 |
| 47 | vaccine study.tw. | 414 |
| 48 | vaccine studies.tw. | 1257 |
| 49 | Vaccines/ | 30838 |
| 50 | vaccin*.tw. | 452266 |
| 51 | Vaccination/ | 113725 |
| 52 | 1 or 2 or 3 or 4 or 5 or 6 or 7 or 8 or 9 or 10 or 11 or 12 or 13 | 4003360 |
| 53 | 14 or 15 or 16 or 17 or 18 or 19 or 20 or 21 or 22 or 23 or 24 or 25 or 26 | 2268960 |
| 54 | 27 or 28 or 29 or 30 or 31 or 32 or 33 or 34 or 35 or 36 or 37 or 38 or 39 or 40 or 41 or 42 or 43 or 44 | 104790 |
| 55 | 45 or 46 or 47 or 48 or 49 or 50 or 51 | 474321 |
| 56 | 52 and 53 and 54 and 55 | 145 |
| **57** | **limit 56 to yr="1990 - 2025"** | **139** |

## Supplementary Table 3. Search results from PsycINFO (Ovid)

| **#** | **Search terms** | **Results** |
| --- | --- | --- |
| 1 | aged.tw. | 286787 |
| 2 | exp Aging/ | 94505 |
| 3 | ageing.tw. | 13925 |
| 4 | elder*.tw. | 74892 |
| 5 | elderly.tw. | 64822 |
| 6 | frail elderly.tw. | 1041 |
| 7 | older people.tw. | 18007 |
| 8 | older adult*.tw. | 72521 |
| 9 | older population.tw. | 2207 |
| 10 | older individual*.tw. | 4445 |
| 11 | oldest old.tw. | 1506 |
| 12 | exp Geriatrics/ | 15205 |
| 13 | geriatric*.tw. | 19635 |
| 14 | very elderly.tw. | 211 |
| 15 | exp Randomized Controlled Trials/ | 1712 |
| 16 | randomi?ed controlled trial.tw. | 33193 |
| 17 | RCT.tw. | 7793 |
| 18 | controlled clinical trial.tw. | 1955 |
| 19 | controlled trial.tw. | 38834 |
| 20 | exp Clinical Trials/ | 14079 |
| 21 | trial*.tw. | 210297 |
| 22 | clinical study.tw. | 2425 |
| 23 | exp Randomized Clinical Trials/ | 591 |
| 24 | randomi?ed.tw. | 116386 |
| 25 | randomi?ation.tw. | 7619 |
| 26 | random allocation.tw. | 320 |
| 27 | randomly allocated.tw. | 4679 |
| 28 | random assignment.tw. | 2102 |
| 29 | vaccine trial.tw. | 125 |
| 30 | vaccine trials.tw. | 162 |
| 31 | vaccine study.tw. | 12 |
| 32 | vaccine studies.tw. | 25 |
| 33 | vaccin*.tw. | 11995 |
| 34 | exp Vaccination/ | 2431 |
| 35 | care home*.tw. | 2532 |
| 36 | exp Nursing Homes/ | 10048 |
| 37 | old age home*.tw. | 149 |
| 38 | aged care home*.tw. | 89 |
| 39 | residential home*.tw. | 600 |
| 40 | residential care.tw. | 4864 |
| 41 | exp Residential Care Institutions/ | 45671 |
| 42 | residential facilit*.tw. | 1480 |
| 43 | exp Long Term Care/ | 7086 |
| 44 | long-term care setting*.tw. | 829 |
| 45 | long-term care facilit*.tw. | 2060 |
| 46 | care home setting*.tw. | 79 |
| 47 | homes for the aged.tw. | 157 |
| 48 | aged care facilit*.tw. | 621 |
| 49 | institutional care.tw. | 1667 |
| 50 | exp Institutionalization/ | 44619 |
| 51 | institutionali?ation.tw. | 5171 |
| 52 | institutionali?ed.tw. | 8505 |
| 53 | 1 or 2 or 3 or 4 or 5 or 6 or 7 or 8 or 9 or 10 or 11 or 12 or 13 or 14 | 446653 |
| 54 | 15 or 16 or 17 or 18 or 19 or 20 or 21 or 22 or 23 or 24 or 25 or 26 or 27 or 28 | 250090 |
| 55 | 29 or 30 or 31 or 32 or 33 or 34 | 12005 |
| 56 | 35 or 36 or 37 or 38 or 39 or 40 or 41 or 42 or 43 or 44 or 45 or 46 or 47 or 48 or 49 or 50 or 51 or 52 | 106335 |
| 57 | 53 and 54 and 55 and 56 | 8 |
| **58** | **limit 57 to yr="1990 -Current"** | **8** |

## Supplementary Table 4. Search results from CINAHL (EBSCOhost)

| [**#**](javascript:__doPostBack('ctl00$ctl00$FindField$FindField$historyControl$ReorderHistoryLink','')) | **Search terms** | **Results** |
| --- | --- | --- |
| S1 | (MH "Aged") | 945,456 |
| S2 | aged | 1,135,614 |
| S3 | (MH "Aging") | 56,967 |
| S4 | aging | 114,016 |
| S5 | ageing | 114,016 |
| S6 | elder | 16,037 |
| S7 | elders | 17,469 |
| S8 | elderly | 113,149 |
| S9 | (MH "Frail Elderly") | 9,079 |
| S10 | frail elderly | 10,304 |
| S11 | older people | 35,810 |
| S12 | older adults | 97,707 |
| S13 | older population | 15,777 |
| S14 | older individuals | 10,969 |
| S15 | oldest old | 2,168 |
| S16 | (MH "Geriatrics") | 6,221 |
| S17 | geriatric | 56,254 |
| S18 | (MH "Aged, 80 and Over") | 345,796 |
| S19 | aged 80 and over | 345,923 |
| S20 | (MH "Randomized Controlled Trials") | 147,767 |
| S21 | randomised controlled trial | 36,640 |
| S22 | rct | 32,519 |
| S23 | controlled clinical trial | 39,511 |
| S24 | controlled trial | 266,351 |
| S25 | (MH "Clinical Trials") | 187,148 |
| S26 | clinical trial | 306,273 |
| S27 | trial | 606,194 |
| S28 | clinical study | 148,351 |
| S29 | randomized | 328,780 |
| S30 | randomised | 59,154 |
| S31 | randomization | 23,262 |
| S32 | randomisation | 21,333 |
| S33 | random allocation | 1,338 |
| S34 | randomly allocated | 12,801 |
| S35 | (MH "Random Assignment") | 89,379 |
| S36 | random assignment | 90,426 |
| S37 | care home | 67,575 |
| S38 | (MH "Nursing Homes") | 26,824 |
| S39 | nursing home | 60,752 |
| S40 | old age home | 3,925 |
| S41 | aged care home | 651 |
| S42 | residential home | 2,360 |
| S43 | residential care | 14,068 |
| S44 | (MH "Residential Facilities") | 5,666 |
| S45 | residential facility | 7,571 |
| S46 | long term care | 43,359 |
| S47 | long term care setting | 2,448 |
| S48 | long term care facility | 35 |
| S49 | care home setting | 2,941 |
| S50 | homes for the aged | 4,926 |
| S51 | aged care facility | 1,626 |
| S52 | institutional care | 5,014 |
| S53 | (MH "Institutionalization") | 2,848 |
| S54 | institutionalisation | 5,120 |
| S55 | institutionalized | 4,904 |
| S56 | vaccine trials | 2,397 |
| S57 | vaccine study | 3,987 |
| S58 | (MH "Vaccines") | 9,686 |
| S59 | vaccines | 78,390 |
| S60 | vaccination | 49,182 |
| S61 | S1 OR S2 OR S3 OR S4 OR S5 OR S6 OR S7 OR S8 OR S9 OR S10 OR S11 OR S12 OR S13 OR S14 OR S15 OR S16 OR S17 OR S18 OR S19 | 1,250,248 |
| S62 | S20 OR S21 OR S22 OR S23 OR S24 OR S25 OR S26 OR S27 OR S28 OR S29 OR S30 OR S31 OR S32 OR S33 OR S34 OR S35 OR S36 | 764,604 |
| S63 | S37 OR S38 OR S39 OR S40 OR S41 OR S42 OR S43 OR S44 OR S45 OR S46 OR S47 OR S48 OR S49 OR S50 OR S51 OR S52 OR S53 OR S54 OR S55 | 158,620 |
| S64 | S56 OR S57 OR S58 OR S59 OR S60 | 94,583 |
| S65 | S61 AND S62 AND S63 AND S64 | 108 |
| **S66** | **S61 AND S62 AND S63 AND S64**  **Limiters- Publication Date: 19900101-20251231** | **107** |

## Supplementary Table 5. Search results from Cochrane Library

| **#** | **Search terms** | **Results** |
| --- | --- | --- |
| #1 | MeSH descriptor: [Aged] in all MeSH products | 283161 |
| #2 | MeSH descriptor: [Frail Elderly] explode all trees | 1191 |
| #3 | MeSH descriptor: [Aged, 80 and over] explode all trees | 72289 |
| #4 | (aging):ti,ab,kw | 19908 |
| #5 | (ageing):ti,ab,kw | 19908 |
| #6 | (elderly):ti,ab,kw | 63404 |
| #7 | (older people):ti,ab,kw | 14371 |
| #8 | (older adults):ti,ab,kw | 28020 |
| #9 | (older population):ti,ab,kw | 13573 |
| #10 | (older individuals):ti,ab,kw | 8093 |
| #11 | (oldest old):ti,ab,kw | 186 |
| #12 | (geriatrics):ti,ab,kw | 1522 |
| #13 | #1 OR #2 OR #3 OR #4 OR #5 OR #6 OR #7 OR #8 OR #9 OR #10 OR #11 OR #12 | 358800 |
| #14 | MeSH descriptor: [Randomized Controlled Trial] explode all trees | 37 |
| #15 | ("randomised controlled trials"):ti | 6068 |
| #16 | ("randomised-controlled trial"):ti | 153340 |
| #17 | (RCT):ti,ab,kw | 43747 |
| #18 | MeSH descriptor: [Controlled Clinical Trial] explode all trees | 40 |
| #19 | (controlled trial):ti | 225976 |
| #20 | MeSH descriptor: [Clinical Trial] explode all trees | 45 |
| #21 | (trial*):ti,ab,kw | 1211719 |
| #22 | MeSH descriptor: [Clinical Study] explode all trees | 45 |
| #23 | (randomized):ti,ab,kw | 1229965 |
| #24 | (randomised):ti,ab,kw | 1229965 |
| #25 | (randomization):ti,ab,kw | 112582 |
| #26 | (randomisation):ti,ab,kw | 112582 |
| #27 | MeSH descriptor: [Random Allocation] explode all trees | 26097 |
| #28 | (randomly allocated):ti,ab,kw | 58048 |
| #29 | (random assignment):ti,ab,kw | 17394 |
| #30 | #14 OR #15 OR #16 OR #17 OR #18 OR #19 OR #20 OR #21 OR #22 OR #23 OR #24 OR #25 OR #26 OR #27 OR #28 OR #29 | 1478384 |
| #31 | (care home*):ti,ab,kw | 34219 |
| #32 | MeSH descriptor: [Nursing Homes] explode all trees | 2140 |
| #33 | (old age home*):ti,ab,kw | 2407 |
| #34 | (aged care home):ti,ab,kw | 12626 |
| #35 | (residential home*):ti,ab,kw | 1245 |
| #36 | (residential care):ti,ab,kw | 2028 |
| #37 | MeSH descriptor: [Residential Facilities] explode all trees | 2676 |
| #38 | (residential facility):ti,ab,kw | 422 |
| #39 | MeSH descriptor: [Long-Term Care] explode all trees | 1553 |
| #40 | (long-term care setting*):ti,ab,kw | 5094 |
| #41 | (long-term care facilit*):ti,ab,kw | 3192 |
| #42 | (care home setting*):ti,ab,kw | 6672 |
| #43 | MeSH descriptor: [Homes for the Aged] explode all trees | 841 |
| #44 | (aged care facilit*):ti,ab,kw | 9055 |
| #45 | (institutional care):ti,ab,kw | 5305 |
| #46 | MeSH descriptor: [Institutionalization] explode all trees | 254 |
| #47 | (institutionalizations):ti,ab,kw | 21 |
| #48 | (institutionalisation*):ti,ab,kw | 159 |
| #49 | (institutionalized):ti,ab,kw | 1388 |
| #50 | (institutionalised):ti,ab,kw | 1388 |
| #51 | #31 OR #32 OR #33 OR #34 OR #35 OR #36 OR #37 OR #38 OR #39 OR #40 OR #41 OR #42 OR #43 OR #44 OR #45 OR #46 OR #47 OR #48 OR #49 OR #50 OR #51 | 55240 |
| #52 | (vaccine trial):ti,ab,kw | 16899 |
| #53 | (vaccine trials):ti,ab,kw | 5602 |
| #54 | (vaccine study):ti,ab,kw | 21344 |
| #55 | MeSH descriptor: [Vaccines] in all MeSH products | 17786 |
| #56 | (vaccin*):ti,ab,kw | 34389 |
| #57 | (vaccines):ti,ab,kw | 19914 |
| #58 | (vaccination):ti,ab,kw | 20116 |
| #59 | #52 OR #53 OR #54 OR #55 OR #56 OR #57 OR #58 | 34431 |
| **#60** | **#13 AND #30 AND #51 AND #59**  **with Cochrane Library publication date from Jan 1990 to Jan 2025** | **224** |

# Appendix 3: Data Extraction Form

| **ADMINISTRATION DETAILS** | |
| --- | --- |
| Study ID | Last name of the first author and publication year |
| Publication status | e.g. full-text paper, conference abstract, trial registration |
| Language | Specify if its non-English language |
| Funding | Public or private funding |
| **STUDY CHARACTERISTICS** | |
| Study title and aim |  |
| Study design | e.g. Randomised Controlled Trial |
| Country | Country/countries where the study was conducted |
| Study population/Study setting | e.g. nursing home residents, long-term care facilities |
| Disease condition |  |
| Study period | Specify start and end date/year |
| Follow up period | Specify length of the follow up period |
| Eligibility Criteria | List of inclusion and exclusion criteria |
| **PARTICIPANT CHARACTERISTICS** | |
| Age (years) | Specify mean/median/range |
| Gender | n (%) for female |
| Race/Ethnicity | n (%) |
| **INTERVENTION AND COMPARATOR** | Details of intervention and comparator/placebo  Route of administration, storage/logistics  Licensed or Investigational vaccine |
| **OUTCOMES REPORTED** | e.g. laboratory, clinical outcomes, adverse events |
| **CARE HOME CHARACTERISTICS** | |
| Number of Care homes | Number of care homes involved/recruited participants |
| Type of care home | e.g. nursing home, residential home |
| Services provided | e.g. personal care, nursing care |
| Size of care home | Number of beds or small/medium/large |
| Ownership | e.g. public or private |
| **QUANTITATIVE DATA: SCREENING AND DROPOUT** | |
| Number screened | n (%) |
| Number recruited | n (%) |
| Number dropped out | n (%) |
| Number of screen failure | n (%) |
| Reasons for screen failure | List of reasons, specify if n (%) reported |
| Reasons for dropout | List of reasons, specify if n (%) reported |
| **QUALITATIVE EVIDENCE OF BARRIERS AND FACILITATORS** | |
| Barriers reported | Challenges faced by investigators in the study: e.g. recruitment/consent/regulatory issues, care home related factors |
| Facilitators reported | Strategies implemented by investigators to overcome the barriers: e.g. recruiting from multiple sites, change of study procedures, collaboration with care homes |
| **ETHICAL AND REGULATORY ASPECTS** | Ethical approval, consent, proxy consent, and incentives provided. Clinical trial registration |

# Appendix 4: Detailed study characteristics of included vaccine trials

| **Author** | **Title** | **Study Population/ Study setting** | **Intervention** | **Control** | **Outcomes reported** |
| --- | --- | --- | --- | --- | --- |
| Treanor et al. 1992^24^ | Protective efficacy of combined live intranasal and inactivated Influenza A virus vaccines in the elderly | Nursing home residents | Monovalent cold-adapted (live attenuated) H3N2 influenza A virus vaccine; [intranasally]; Unlicensed | Placebo: sterile veal infusion broth [intranasal] | Antibody response and adverse events |
| Remarque et al. 1993^25^ | Improvement of the immunoglobulin subclass response to influenza vaccine in elderly nursing-home residents by the use of high-dose vaccines | Nursing home residents and young volunteers | Trivalent subunit influenza vaccine [intramuscularly]; Unlicensed | No placebo group | Antibody response |
| Palache et al. 1993^26^ | Antibody response after influenza immunization with various vaccine doses: a double-blind, placebo- controlled, multi-centre, dose-response study in elderly nursing-home residents and young volunteers | Nursing home residents and young volunteers | Trivalent influenza subunit vaccine [intramuscular]; Unlicensed | Placebo: 0.5 ml saline [intramuscular] | Antibody response and adverse events |
| Glück et al. 1994^27^ | Immunogenicity of new virosome influenza vaccine in elderly people | Nursing home residents | A trivalent virosome influenza vaccine [intramuscular]; Unlicensed | Inflexal, whole virus OR Influvac-92, subunit vaccine [intramuscular]; Licensed | Antibody response and adverse events |
| Gravenstein et al. 1994 ^28^ | Efficacy of an Influenza Hemagglutinin-Diphtheria Toxoid Conjugate Vaccine in Elderly Nursing Home Subjects During an Influenza Outbreak | Nursing home residents | Trivalent influenza hemagglutinin-diphtheria toxoid conjugate vaccine [intramuscular]; Unlicensed | Fluzone: Trivalent influenza hemagglutinin-subunit vaccine [intramuscular]; Licensed | Antibody response and clinical efficacy (respiratory illness) |
| Gorse et al. 1996^29^ | Induction of mucosal antibodies by Live attenuated and inactivated influenza virus vaccines in the chronically ill elderly | Nursing home residents | Trivalent inactivated subvirion influenza vaccine [intramuscularly] AND Bivalent cold-recombinant live attenuated influenza A vaccine [intranasal]; Unlicensed | Trivalent inactivated subvirion influenza vaccine [intramuscularly] AND Saline placebo [intranasal]; Licensed | Antibody response |
| Gauthey et al. 1996^30^ | Side effects of influenza vaccination among elderly persons | Long-term care facilities & Nursing homes | Inflexal®: Trivalent inactivated whole virus flu vaccine; Licensed | Influvac®: Trivalent subunit flu vaccine; Licensed | Adverse events |
| Rudenko et al. 2001^31^ | Immunogenicity and efficacy of Russian live attenuated and US inactivated influenza vaccines used alone and in combination in nursing home residents | Elderly or chronically ill nursing home residents | US trivalent inactivated split-virus influenza vaccine, [intramuscular]; Licensed and/or Russian trivalent live attenuated cold-adapted influenza vaccine [intranasal]; Unlicensed | Sterile physiological saline [intramuscular] and/or Lyophilized allantoic fluid [intranasal] | Antibody response and clinical efficacy (surveillance for respiratory illness) |
| Roos-van Eijndhoven et al. 2001^32^ | Randomized Controlled Trial of Seroresponses to Double Dose and Booster Influenza Vaccination in Frail Elderly Subjects | Residents of long-term care facilities | Trivalent split virus influenza vaccine for the 1997-1998 influenza season [intramuscular]; Licensed | Placebo: phosphate buffered saline OR booster dose of trivalent split virus influenza vaccine | Antibody response |
| Pregliasco et al. 2001 ^33^ | Immunogenicity and safety of three commercial influenza vaccines in institutionalized elderly | Elderly nursing home residents | Fluad: adjuvanted subunit vaccine with MF59-adjuvant OR Inflexal Berna: inactivated whole virus vaccine OR Inflexal V Berna: virosome subunit vaccine [intramuscular]; Licensed | Three commercial influenza vaccines each containing three influenza strains for the 1998-1999 season were compared | Antibody response and clinical efficacy (surveillance for respiratory illness) and adverse events |
| Baldo et al. 2001^34^ | Comparison of three different influenza vaccines in institutionalised elderly | Nursing home residents | Fluad®: MF59-adjuvanted subunit virus vaccine OR Inflexal-V®: Virosomal vaccine OR Mutagrip®: Split virus vaccine [intramuscular]; Licensed | Three commercial influenza vaccines were compared | Antibody response and adverse events |
| Ben-Yehuda et al. 2003^35^ | Immunogenicity and safety of a novel IL-2-supplemented liposomal influenza vaccine (INFLUSOME-VAC) in nursing-home residents | Nursing home residents | INFLUSOME-VAC: interleukin-2 (IL-2) -supplemented trivalent liposomal influenza vaccine, [intramuscular]; Unlicensed | Vaxigrip: Trivalent split virion vaccine (purified virus vaccine) [intramuscular]; Licensed | Antibody response and adverse events |
| Muszkat et al. 2003^36^ | Local and systemic immune response in nursing-home elderly following intranasal or intramuscular immunization with inactivated influenza vaccine | Elderly nursing home residents | Novel inactivated trivalent whole influenza virus vaccine [Intranasal, twice 21 days apart]; Unlicensed | Inactivated trivalent split influenza vaccine [intramuscular]; Licensed | Antibody response and adverse events |
| Valenzuela et al. 2007^37^ | Immunogenicity of a 23-valent pneumococcal polysaccharide vaccine in elderly residents of a long-term care facility | Elderly nursing home residents | Pneumo 23™: 23-valent pneumococcal polysaccharide [intramuscular]; Licensed | Tetavax^TM^: Monovalent tetanus toxoid vaccine [intramuscular]; Licensed | Antibody response and adverse events |
| Gaughran et al. 2007^38^ | Flu: Effect of vaccine in elderly care home residents: A Randomized Trial | Elderly permanent care home residents | Trivalent influenza vaccine and a booster trivalent influenza vaccine (same brand) [intramuscular]; Licensed | Trivalent influenza vaccine [intramuscular] and no booster influenza vaccine; Licensed | Antibody response and clinical outcomes (hospitalisation, death) |
| Maruyama et al. 2010^39^ | Efficacy of 23-valent pneumococcal vaccine in preventing pneumonia and improving survival in nursing home residents: double blind, randomised and placebo-controlled trial | Nursing home residents from hospital affiliated nursing homes | Pneumovax: 23-valent pneumococcal polysaccharide vaccine [Intramuscular]; Licensed | Placebo: Sodium chloride [Intramuscular] | Clinical outcomes (incidence of pneumonia and death) |
| Chan et al. 2014^40^ | Immunogenicity and safety of intradermal trivalent influenza vaccination in nursing home older adults: A Randomized Controlled Trial | Nursing home older adults | Intanza: single full-dose (15 µg) intradermal trivalent influenza vaccine; Licensed | Vaxigrip: single full-dose (15 µg) intramuscular trivalent influenza vaccine; Licensed | Antibody response and adverse events |
| Namkoong et al. 2015^41^ | Comparison of the immunogenicity and safety of polysaccharide and protein-conjugated pneumococcal vaccines among the elderly aged 80 years or older in Japan: An open-labelled randomized study | Nursing home residents | Prevenar (PCV7): 7-valent pneumococcal protein-conjugated vaccine [subcutaneous]; Unlicensed for use in older adults | Pneumovax; 23-valent pneumococcal polysaccharide vaccine [subcutaneous]; Licensed | Antibody response and adverse events |
| Nace et al. 2015 ^42^ | Randomized, Controlled Trial of High-Dose Influenza Vaccine Among Frail Residents of Long-Term Care Facilities | Frail elderly residents of long-term care facilities | Fluzone: High dose inactivated trivalent influenza vaccine, [intramuscular]; Licensed | Fluzone: Standard dose inactivated trivalent influenza vaccine [intramuscular]; Licensed | Antibody response and Serious Adverse Events (death) |
| Didion et al. 2025^43^ | Which Enhanced Influenza Vaccine Has the Greatest Immunogenicity in Long-Term Care Residents: The Adjuvanted or the High-Dose Formulation? | Long-term care facility residents | Fluad: MF59 adjuvanted trivalent inactivated flu vaccine [intramuscular]; Licensed | Fluzone: High dose trivalent inactivated flu vaccine [intramuscular]; Licensed | Antibody response |

**NR:** Not Reported. **Note:** The term 'elderly' is used only where it reflects the terminology of the original study authors, and the review team recognise that this is not the preferred terminology for referring to older people.
